# Engineered *Yarrowia lipolytica* for sustainable heavy metal bioremediation via enhanced alkaline phosphatase-driven biomineralization

**DOI:** 10.1016/j.engmic.2026.100310

**Published:** 2026-10-23

**Authors:** Zhen Zheng, Jianping Liu, Xiaoman Xie, Xinyi Hu, Wanlin Zheng, Shuhan Dai, Qiyang Wang, Yuan Liao, Jinyong Yan, Min Yang, Yunjun Yan, Li Xu

**Affiliations:** a Key Laboratory of Molecular Biophysics of Ministry of Education, College of Life Science and Technology, Huazhong University of Science and Technology, Wuhan, China; b Geological Environmental Center of Hubei Province, Hubei Geological Bureau, Wuhan 430074, China

**Keywords:** Alkaline phosphatase (PhoX), Bio-mineralization, Heavy metal bioavailability, *Yarrowia lipolytica*, Metal immobilization, Pollution remediation

## Abstract

Heavy metal contamination poses persistent risks owing to metal mobility and bioavailability. In this study, we explored a biological strategy for altering the fate of metal ions by engineering the yeast *Yarrowia lipolytica* to secrete alkaline phosphatase (PhoX), thereby driving enzymatic metal-phosphate precipitation. Through copy number optimization and process refinement, a recombinant strain was obtained harboring three copies of *phoX*, exhibiting high extracellular PhoX activity (136.67 U/mL), and it showed a broad-spectrum precipitation of metal ions, including Cd^2+^, Mn^2+^, Co^2+^, Cr^3+^, Cu^2+^, Ni^2+^ and Pb^2+^. A two-step process was then designed, achieving near-quantitative removal efficiencies (>90%) for Cd^2+^, Mn^2+^, Co^2+^, Cr^3+^, and Pb^2+^ (ranging from 90.92% to 99.28%), while Cu^2+^ (89.09%) and Ni^2+^ (78.35%) showed slightly lower efficiencies. The system also achieved high removal capacities of up to 1251.64 mg Cd^2+^/g biomass. Microscopic and crystallographic analyses confirmed the formation of cell-associated, crystalline metal phosphate minerals, with Co^2+^ immobilized as Co_3_(PO_4_)_2_·8H_2_O. By utilizing a generally recognized as safe microorganism to convert soluble, bioavailable metals into stable mineral phases, we elucidated a biologically mediated immobilization pathway that can reduce metal toxicity and mobility, offering a sustainable mechanism for remediating metal-contaminated environments.

## Highlights

• Engineered *Yarrowia lipolytica* alters the environmental fate of heavy metals.
• A stepwise bioprocess removed >99% of Cd^2+^ and Pb^2+^ from solution.
• Ultrahigh removal capacity reached 1251.64 mg Cd^2+^/g of biomass.
• Biomineralization converted metals into stable crystalline phosphate minerals.
• The data provide a biological pathway to reduce metal bioavailability and mobility.

## Graphical abstract

**Figure:**
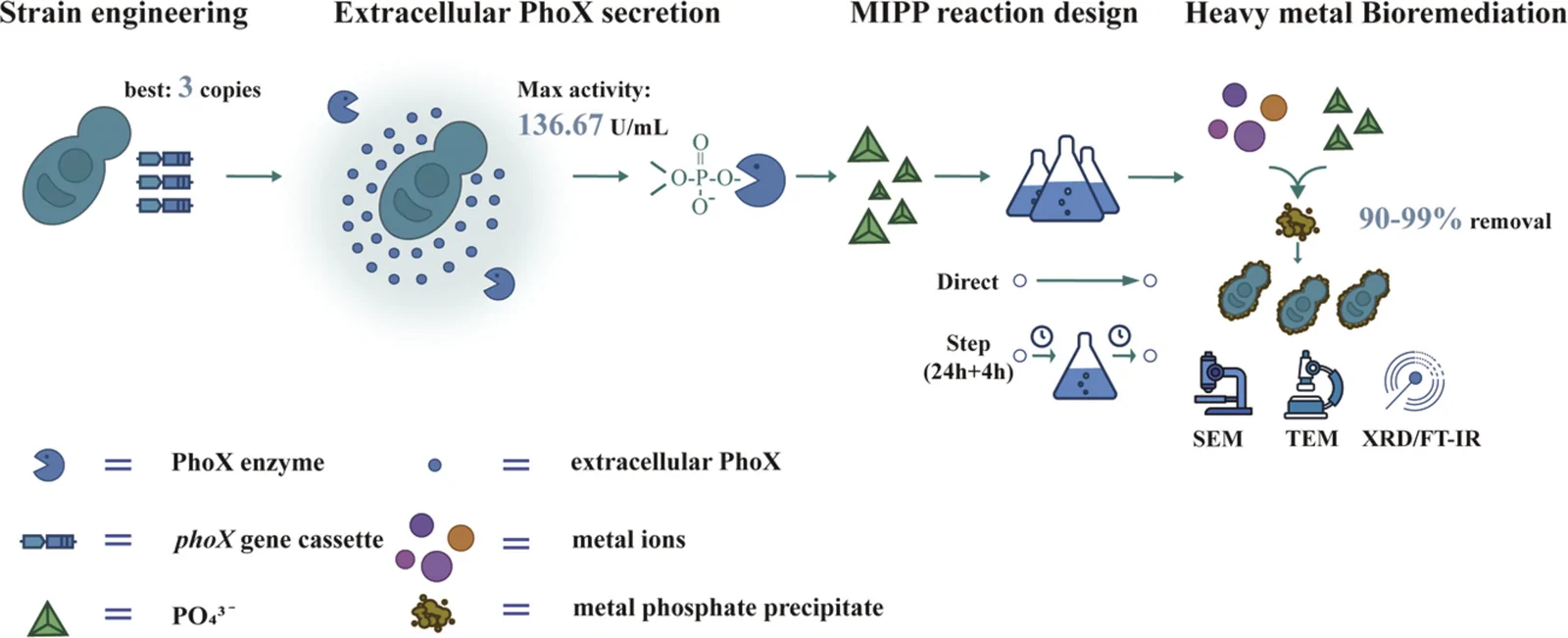

## 1 Introduction

The pervasive contamination of soil and aquatic ecosystems by heavy metals poses threats to environmental health owing to their toxicity, mobility, and bioavailability [[1], [2], [3], [4]]. Effective remediation requires strategies that not only remove metals but also alter their speciation to reduce ecological risks. Conventional physicochemical methods often entail high resource consumption and can generate secondary waste, highlighting the need for sustainable, biologically driven approaches that target the environmental fate of metal pollutants [[5], [6], [7], [8]]. Among biological strategies, microbially induced mineralization offers a pathway to convert soluble, bioavailable metal ions into stable solid phases, thereby reducing their mobility and toxicity [[9], [10], [11], [12], [13], [14], [15]]. Although microbially induced carbonate precipitation (MICP) has been widely studied, its reliance on urea hydrolysis produces ammonia, thus creating secondary pollution and limiting its environmental compatibility [[16], [17], [18], [19]]. In contrast, microbially induced phosphate precipitation (MIPP) represents a more environmentally congruent process that facilitates the formation of highly stable metal phosphate minerals with low solubility, enabling long-term sequestration with minimal risk of remobilization [[14], [20], [21], [22], [23]]. This enzymatic conversion of metal species addresses the core challenge in metal pollution of controlling bioavailability through speciation changes. The practical implementation of MIPP relies on robust, environmentally relevant microbial catalysts. The generally recognized as safe (GRAS) yeast *Yarrowia lipolytica* is a promising host because of its inherent stress tolerance, genetic tractability, and capacity for secretory protein production [24,25]. Its physiological resilience in metal-contaminated environments makes it suitable for developing biocatalytic platforms for altering the fates of metals in situ [[26], [27], [28], [29], [30]}.

A key enzyme enabling efficient phosphate release for MIPP is alkaline phosphatase (ALP, EC 3.1.3.1) that hydrolyzes organic phosphates to inorganic phosphate [22]. Although ALP activity has been explored in bioremediation contexts, its practical application for large-scale metal immobilization has been limited by low production yields, particularly in eukaryotic expression systems [31,32]. Engineering *Y. lipolytica* for high-level extracellular secretion of ALP represents a critical step in developing an efficient biological process for environmental remediation via metal-phosphate mineralization.

This study was designed to elucidate and enhance a biological pathway for metal immobilization by engineering *Y. lipolytica* for high-level secretion of the alkaline phosphatase, PhoX. We systematically optimized enzymatic production, evaluated the bio-mineralization efficiency and metal-removal capacity of engineered *Y. lipolytica* strains against a spectrum of heavy metal ions (Cd^2+^, Mn^2+^, Co^2+^, Cr^3+^, Cu^2+^, Ni^2+^, and Pb^2+^), and characterized the resulting mineral products. By focusing on the conversion of soluble metals into stable phosphate minerals, we generated data that provides mechanistic insights into a microbially facilitated immobilization process that reduces metal bioavailability, offering a sustainable biological strategy for mitigating heavy metal contamination in water and soil environments.

## 2 Methods and materials

### 2.1 Plasmids, strains, and recombinant strain construction

A codon-optimized *phoX* gene (GenBank: LC157911.1) was synthesized by Wuhan Tianyi Huayu Gene Technology Co., Ltd. (Sequence S1) and subcloned into the pUC57 vector to generate plasmid pUC57-*phoX*. This gene was subsequently amplified via PCR using pUC57-*phoX* as the template and primers F_pUC_57-*phoX*-7166 and R_pUC57-*phoX*-7166 as the primer pair. The plasmid pUAxp7166-rol was double-digested with *Sfi* I and *Cla* I, and the linearized fragment was gel-purified. The purified *phoX* PCR product was ligated into this digested vector using homologous recombination. The resulting construct was transformed into *Escherichia coli* Top10F ′ competent cells. Positive clones were screened by colony PCR using primers F_ *phoX*-2–7166 and R_*phoX*-2–7166, and plasmid DNA was extracted and verified by DNA sequencing. A confirmed single-copy expression plasmid was designated as pUAxp7166-*phoX*.

Multi-copy plasmids were constructed using the isocaudamer sites *Spe* I/*Nhe* I and *Nsi* I) flanking the expression cassette in pUAxp7166. Sequential digestion and ligation steps produced plasmids harboring tandem copies of the *phoX* cassette. Plasmids pUAxp7166–2*phoX* (two copies), Po1h-3*phoX* (three copies)*,* Po1h-4*phoX* (four copies), Po1h-5*phoX* (five copies), and Po1h-6*phoX* (six copies) were verified by restriction digestion.

Plasmids were linearized with *Nsi* I and separately transformed into *Y. lipolytica* Po1h competent cells via the lithium acetate method. Transformants were selected on minimal dextrose liquid medium (13.4 g/L yeast nitrogen base (YNB), 0.4 mg/L biotin, and 20 g/L glucose). The Ura3 selection marker was subsequently excised via culturing on oleic acid-containing medium (13.4 g/L YNB, 0.4 mg/L biotin, 0.027 g/L uracil, 20 mL/L oleic acid) [33]. Genomic integration and copy numbers were confirmed by polymerase chain reaction (PCR). This process generated the engineered strains Po1h-1phoX*,* Po1h-2phoX, Po1h-3phoX*,* Po1h-4phoX, Po1h-5phoX*,* and Po1h-6phoX.

All engineered strains and the control Po1h strain were maintained on yeast extract peptone dextrose medium (YPD) agar plates (10 g/L yeast extract, 20 g/L peptone, and 20 g/L glucose, 20 g/L agar).

### 2.2 Fermentation and culture optimization

For pre-culture, a single colony was inoculated into YPD broth and incubated overnight. For the main fermentation, a two-stage process was used, involving subsequent expansion in BMSY medium (50 g/L d-sorbitol, 10 g/L yeast extract, 20 g/L tryptone, 100 mM potassium phosphate buffer pH 6.8, 13.4 g/L YNB and 0.4 mg/L biotin).

Optimization of culture conditions for the Po1h-3phoX strain was performed by systematically varying the carbon source type (sorbitol, sucrose, glucose, glycerol), carbon concentration (3–7% m/v), inoculation density (1–5% v/v), initial medium pH (5.5–7.5), and culture volume (30–70 mL in a 500 mL baffled flask).

### 2.3 Co-expression of helper proteins

To potentially enhance PhoX secretion, helper-protein genes (*vgb, pdi, kar2*, and *hac1*) were individually cloned into the integrative vector pUXpr7166. The resulting plasmids pUXpr7166-vgb, pUXpr7166-pdi, pUXpr7166-kar2, and pUXpr7166-hac1 were linearized with *Nsi* I and integrated into the genome of the Po1h-3phoX strain, generating the recombinant strains Po1h-3phoX-vgb, Po1h-3phoX-pdi, Po1h-3phoX-kar2, and Po1h-3phoX-hac1. Correct genomic integration was verified by PCR. Enzyme activities after a 5-day fermentation were compared with that of the parental strain. Relative enzyme activities were calculated, with the highest enzyme activity set to 100%.

### 2.4 Enzyme activity assay

PhoX activity in the culture supernatant was measured using a commercial PhoX Activity Assay Kit (Beijing Boxbio Science & Technology Co., Ltd., China) according to the manufacturer’s instructions. One unit (U) of enzyme activity was defined as the amount of enzyme that catalyzed the formation of 1 μmol of phenol per min at 37 °C and pH 10.0.

The extracellular protein concentrations in culture supernatants were determined by performing bicinchoninic acid protein assays with bovine serum albumin as the standard. Because the medium contained proteinaceous components, uninoculated medium was measured as the medium blank. For each sample, the measured protein concentration was first corrected by its dilution factor, and the medium blank was then subtracted. Extracellular protein concentrations were calculated as: protein assay value × dilution factor − medium blank. Protein-normalized PhoX activity was calculated by dividing extracellular PhoX activity (U/mL) by extracellular protein concentration (mg/mL) and expressed as U/mg extracellular protein. The optical density at 600 nm (OD_600_) of each fermentation culture was measured after appropriate dilution, and the biomass-normalized PhoX activity was calculated as the extracellular PhoX activity divided by the OD_600_ and expressed as U/OD_600_. These normalized values were calculated separately for each biological replicate before averaging.

### 2.5 Metal ion phosphate precipitation assay

#### 2.5.1 Reaction setup

Fermentation broths of the control strain (Po1h) and the engineered strain (Po1h-3phoX) were centrifuged, and the cell pellets were resuspended in fresh buffer to an OD_600_ of 0.8–1.0. A standard 10 mL MIPP reaction system contained 8.9 mL of diluted broth, 1 mL of 0.5 M *β*-sodium glycerophosphate (as the phosphate donor substrate), and 100 μL of a 1 M stock solution of the target metal ion, yielding a final metal ion concentration of 10 mM. All reactions were conducted at 37 °C with rotation at 200 rpm.

Two distinct operational modes were employed to evaluate biomineralization efficiency. In the direct reaction method, the cell suspension, substrate, and metal ion solution were combined simultaneously and incubated for either 2 or 4 h. In contrast, the stepwise reaction method involved a 24 h pre-incubation of the cell suspension with the substrate to enable enzymatic generation of inorganic phosphate prior to metal exposure, after which the metal ion solution was added, and the reaction continued for an additional 4 h.

#### 2.5.2 Biomineralization kinetic analysis

To evaluate the time-dependent behavior of the MIPP process, we selected Co^2+^ as a representative metal ion for biomineralization kinetic analysis. The reaction system was prepared as described in Section 2.5.1 using Po1h-3phoX as the engineered strain, β-glycerophosphate as the phosphate donor, and Co^2+^ at a final concentration of 10 mM. Reactions were conducted at 37 °C with shaking at 200 rpm. Samples were collected at 0, 15, 30, 60, 120, 240, and 360 min, followed by centrifugation at 5000 rpm and 4 °C for 7 min. The residual Co^2+^ concentration in the supernatant was determined by inductively coupled plasma-optical emission spectrometry (ICP-OES) after acid digestion, and the Co^2+^-removal efficiency at each time point was calculated as described in Section 2.5.3.

The kinetic data were fitted using a pseudo-second-order kinetic model, expressed as:

(1) $$\begin{array}{c} \frac{t}{q_{t}}=\frac{1}{k_{2}q_{e}^{2}}+\frac{t}{q_{e}} \end{array}$$

where q_t_ and q_e_ represent the amount of Co^2+^ removed at time *t* and at equilibrium (mg/g), respectively, and *k*_2_ is the pseudo-second-order rate constant (g·mg⁻¹·min⁻¹). The kinetic parameters were obtained from the slope and intercept of the linearized plot (*t*/*q*_t_​ versus *t*).

#### 2.5.3 Metal analysis and removal calculation

Post-reaction, samples were centrifuged at 5000 rpm and 4 °C for 7 min. The supernatant was digested by boiling with 5 mL of 6 M HNO_3_ for 25 min, filtered through a 0.22 μm membrane, and diluted with HNO_3_ to a final acid concentration of 3%–5%. Metal ion concentrations were determined using an ICP-OES instrument (iCAP7200plus, Thermo Fisher Scientific). Standard curves (1–7.5 mM) were prepared for each metal.

The metal-removal efficiency (*R*, %) and metal-removal capacity (*Q*, mg/g) were calculated using Eqs. (2) and (3):

(2) $$\begin{array}{c} R=\frac{C_{i}-C_{f}}{C_{i}}\times 100\% \end{array}$$

(3) $$\begin{array}{c} Q=\frac{\left(C_{i}-C_{f}\right)\times M_{w}}{OD_{600}\times E\times 1000} \end{array}$$

where, *C_i_* and *C_f_* are the initial and final metal concentrations (μM), respectively; *M_w_* is the atomic weight of heavy metal; OD_600_ is as defined above; and *E* is the average cell dry weight per OD_600_ unit in 1 L, determined experimentally as described below.

For dry cell weight (DCW) determinations, cell cultures with different OD_600_ values were harvested by centrifugation, washed twice with distilled water, dried to constant weight at 60 °C for 72 h, and weighed to establish an OD_600_–DCW calibration curve. The resulting conversion factor *E* (g DCW/L per OD_600_ unit) was used for biomass normalization in removal‑capacity calculations.

For routine DCW measurements, the cell pellet from 8.9 mL of diluted culture (OD_600_ = 0.8–1.0) was collected by centrifugation at 8000 rpm and 4 °C for 10 min, washed twice with distilled water, and dried to constant weight at 60 °C for 72 h.

### 2.6 Characterization of MIPP products

#### 2.6.1 Electron microscopy

The morphological features of the mineralized precipitates were examined using complementary electron microscopy techniques. For scanning electron microscopy (SEM), freeze-dried MIPP samples were mounted on conductive adhesive, sputter-coated with gold for 180 s, and imaged using a GeminiSEM 300 microscope (Carl Zeiss). For transmission electron microscopy (TEM), pelleted materials were fixed in glutaraldehyde, deposited on copper grids, air-dried, and observed using an HT7700 microscope (Hitachi).

#### 2.6.2 Crystallographic and spectroscopic analysis

Crystallographic phase composition and molecular functional groups were analyzed to characterize the mineral products. X-ray diffraction (XRD) analysis was performed using freeze-dried powders, with patterns collected from 10° to 90° (2θ). Phase identification was conducted using MDI Jade 6.0 software. Fourier transform infrared spectroscopy (FT-IR) was carried out by mixing approximately 1–2 mg of freeze-dried sample with KBr, pressing the mixture into transparent pellets, and analyzing the absorption spectra to identify characteristic functional groups involved in the mineralization process.

### 2.7 Statistical analysis

All experiments were performed with three independent biological replicates (*n* = 3). Data are presented as mean ± standard deviation (SD). Statistical analysis was conducted using one‑way analysis of variance followed by Tukey’s post‑hoc test. Significance levels are indicated in the figures as follows: NS (not significant, *P* > 0.05), * (significant, *P* < 0.05), and ^⁎⁎^ (highly significant, *P* < 0.01).

## 3 Results

### 3.1 Shake flask fermentation and heterologous expression of PhoX in Y. lipolytica

A recombinant *Y. lipolytica* strain was constructed harboring a single copy of the *phoX* gene. The strain was cultivated in a 500 mL baffled flask. After 120 h of fermentation, the whole-cell broth exhibited a PhoX activity of 7.52 U/mL (Fig. 1a). To test if the recombinant PhoX enzyme could still be a secreted enzyme, the culture was centrifuged, yielding the supernatant and the cell pellet. Examination of the PhoX activity in two fractions revealed that the enzymatic activity was primarily detected in the supernatant with negligible residual activity retained in the cell extracts. Thus, the heterologously expressed PhoX was also successfully secreted into the culture media, enabling the use of whole-cell broth for subsequent applications without the need for cell disruption.

**Fig. 1:**
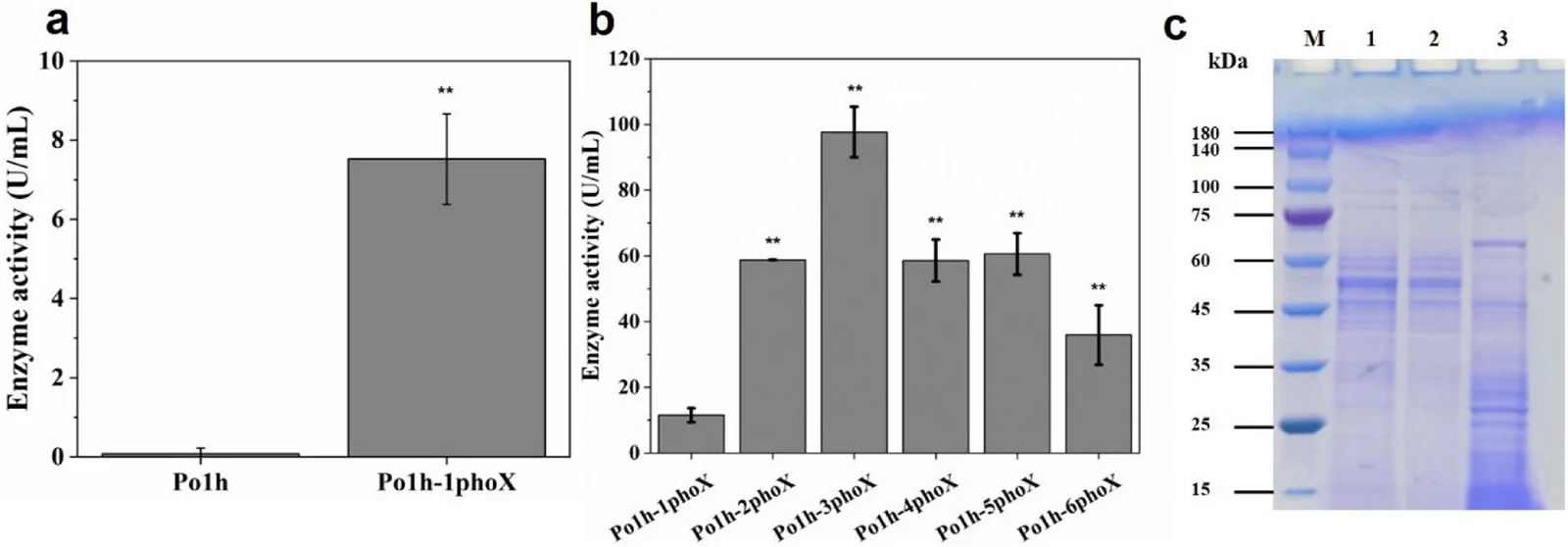
Enzyme activity and SDS-PAGE analysis of engineered strains. (a) Enzyme activity of a single-copy *phoX* engineered strain. (b) Enzyme activity of engineered strains with one to six copies of *phoX*. (c) SDS-PAGE analysis of extracellular proteins in culture supernatants. Lane M, protein marker; Lane 1, wild-type *Y. lipolytica* Po1h supernatant (control); Lane 2, empty-vector control supernatant; Lane 3, Po1h-3phoX culture supernatant. For (a) and (b), data are presented as the mean ± SD (*n* = 3). Asterisks indicate statistically significant differences among groups: *P < 0.05, **P < 0.01; NS, not significant (*P* > 0.05) (one-way ANOVA followed by Tukey’s post-hoc test).

To optimize the enzyme production, recombinant strains carrying one to six copies of the *phoX* gene were constructed and fermented. PhoX activity increased with the gene copy number up to three copies, reaching a maximum of 97.50 U/mL, which was 12.97-fold higher than that of the single-copy strain (Fig. 1b). Beyond three copies, activity significantly decreased, likely due to the metabolic burden associated with excessive heterologous gene expression. These results demonstrate a clear gene dosage effect, with an optimal gene copy number of three for maximizing PhoX yield in this host.

The expression and secretion of PhoX were further evaluated by sodium dodecyl sulfate–polyacrylamide gel electrophoresis (SDS-PAGE) analysis of extracellular protein fractions. Compared with the wild-type strain and empty-vector control, the engineered Po1h-3phoX strain showed an enhanced extracellular protein band near the predicted molecular weight of PhoX (Fig. 1c). In addition, a protein region near the predicted molecular weight of PhoX from the enriched extracellular fraction was excised from the SDS-PAGE gel and subjected to mass spectrometric analysis, confirming the presence of PhoX with 39.9% sequence coverage (Fig. S1). These results support the extracellular secretion of recombinant PhoX from *Y. lipolytica.*

To distinguish volumetric extracellular activity from biomass- and extracellular protein-related effects, the OD_600_ and extracellular protein concentration were measured for the optimized multi-copy strains, and normalized PhoX activities were calculated (Table S4). Po1h-3phoX showed the highest volumetric PhoX activity (124.12 ± 5.14 U/mL) and biomass-normalized activity (3.80 ± 0.53 U/OD_600_) among the tested strains with different copy numbers, although its OD_600_ (33.00 ± 3.77) was not higher than that of Po1h-1phoX (38.50 ± 2.29) or Po1h-4phoX (34.17 ± 3.88). In addition, Po1h-3phoX maintained a high extracellular protein-normalized activity of 53.30 ± 6.03 U/mg extracellular protein, comparable with that of Po1h-4phoX (56.72 ± 7.37 U/mg). Because Po1h-3phoX exhibited the highest volumetric and biomass-normalized activities, it was selected as the best-performing strain for subsequent MIPP experiments. These results indicated that the increased extracellular PhoX activity in Po1h-3phoX was not only attributable to biomass accumulation and the total extracellular protein concentration but also reflected a higher protein-normalized PhoX activity, compared with the low-copy strains (Po1h-1phoX and Po1h-2phoX).

### 3.2 Systematic optimization of culture conditions for enhanced PhoX production

To develop a scalable and efficient process, key fermentation parameters were optimized using the Po1h-3phoX strain. Among the carbon sources tested (sorbitol, sucrose, glucose, and glycerol), sorbitol was uniquely effective, supporting high PhoX activity, whereas others yielded negligible production (Fig. 2a). This specificity might have been linked to *Y. lipolytica*’s native metabolism and the impact of carbon catabolite repression on heterologous expression.

**Fig. 2:**
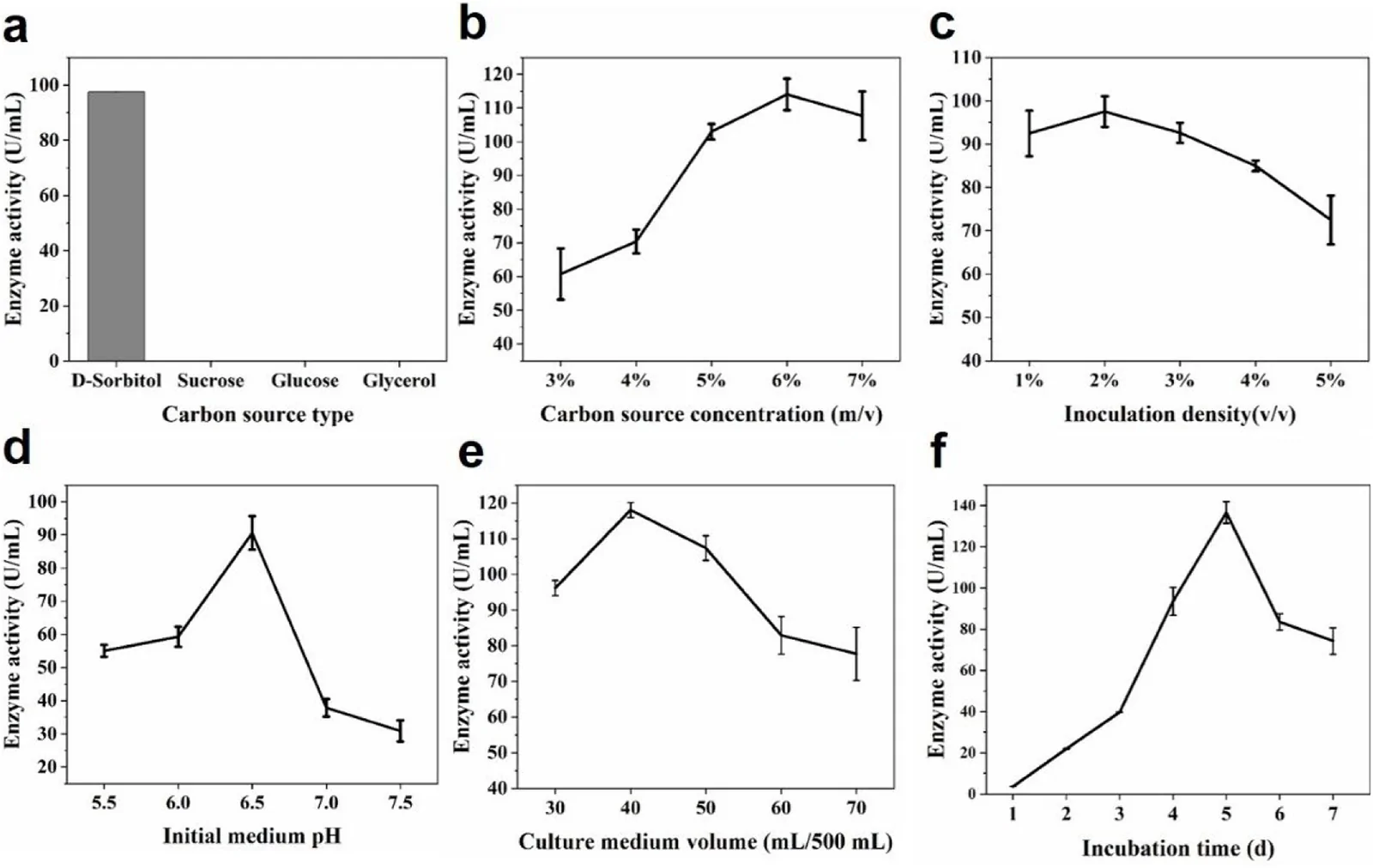
Optimization of culture conditions for the engineered Po1h-3phoX strain. (a) Carbon source type. (b) Carbon source concentration. (c) Inoculum density. (d) Initial pH of medium. (e) Culture medium volume. (f) Incubation time. Data are presented as the mean ± SD (*n* = 3).

Subsequent optimization revealed the following ideal conditions: 6% (m/v) sorbitol concentration (Fig. 2b), a 2% inoculum density (Fig. 2c), an initial medium pH of 6.5 (Fig. 2d), and a culture volume of 40 mL in a 500 mL baffled flask (Fig. 2e). Applying this combined strategy increased the PhoX activity to 136.67 U/mL on day 5, representing a 1.40–fold enhancement over pre-optimized conditions (Fig. 2f). This optimization not only boosted the yield but also provided a foundation for a sustainable process scaling by defining resource-efficient parameters.

Given the role of auxiliary proteins in facilitating protein folding and secretion in yeast, we co-expressed genes encoding Vgb (vitreoscilla hemoglobin), Pdi (protein disulfide isomerase), Kar2 (ER chaperone), and Hac1 (UPR transcription factor). However, none of these co-expressed genes improved PhoX activity (Fig. S2). Strains expressing Pdi, Kar2, or Hac1 showed reduced yields.

### 3.3 Engineered Y. lipolytica for metal ion phosphate precipitation: performance and characterization

#### 3.3.1 Establishing a biologically relevant MIPP assay

Initial MIPP assays performed using standard BMSY medium, which contains phosphate buffer, showed high Co^2+^ precipitation for both control and engineered strains (>72%), likely due to chemical precipitation (Fig. S3). When potassium phosphate was replaced with water, the mineralization rate for the engineered strain (Po1h-3phoX-H_2_O) remained high (80.95%), whereas that of the control strain (Po1h-H_2_O) decreased to 33.69%. These results confirm that the observed MIPP activity in the engineered strain was primarily enzymatically driven. All subsequent MIPP experiments were therefore conducted in phosphate-free medium to accurately assess biological mineralization.

#### 3.3.2 Broad-spectrum metal biomineralization capability

The engineered strain was tested against a panel of toxic divalent and trivalent metal ions (Cd^2+^, Mn^2+^, Co^2+^, Cr^3+^, Cu^2+^, Ni^2+^, and Pb^2+^). Visual inspection after a 2 h reaction revealed abundant, colored precipitates in tubes containing the engineered strains, contrasting with the minimal precipitation observed with the control (except for Pb^2+^ and Cu^2+^) (Fig. 3).

**Fig. 3:**
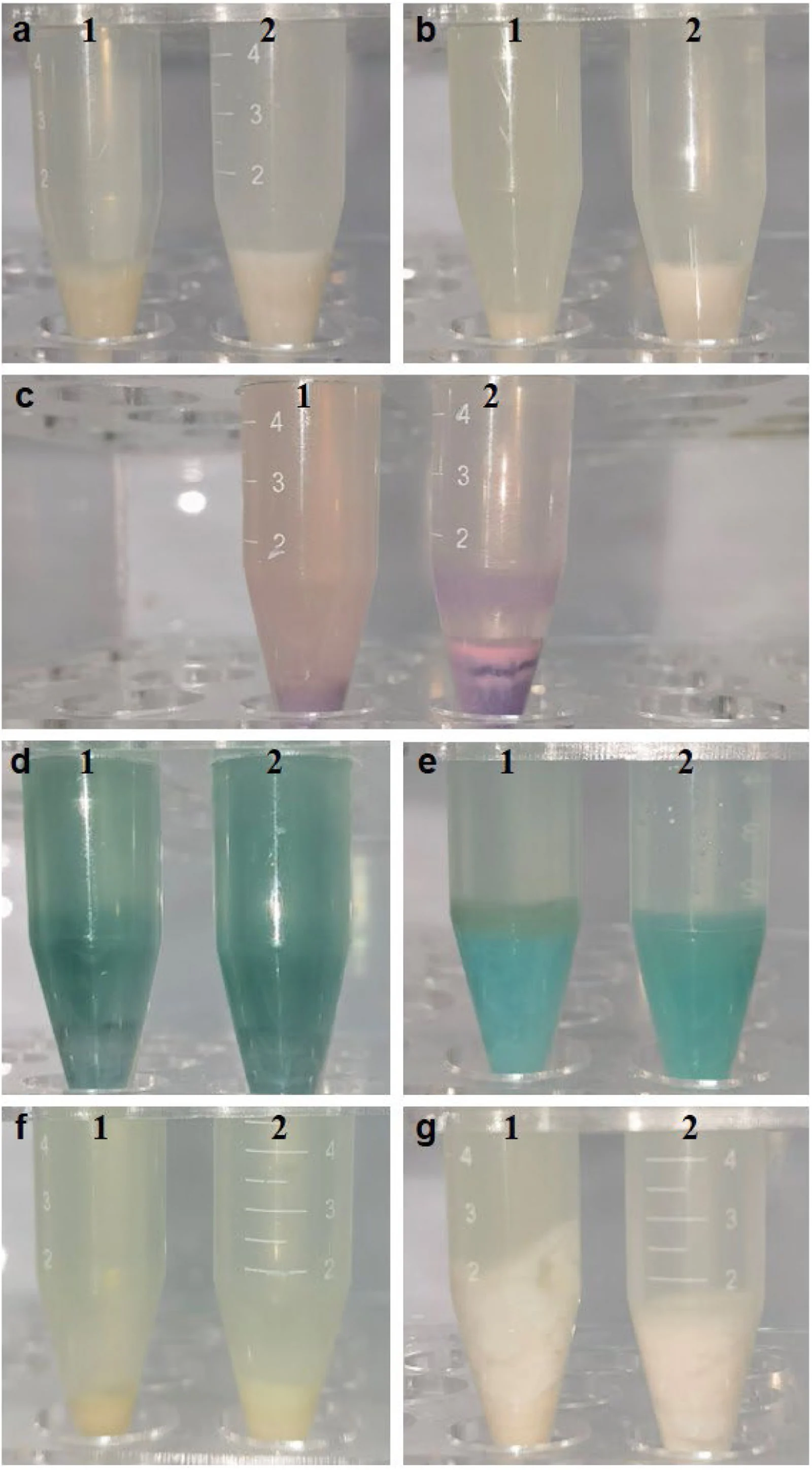
Visual assessment of MIPP reaction effects. (a) Cd^2+^, (b) Mn^2+^, (c) Co^2+^, (d) Cr^3+^, (e) Cu^2+^, (f) Ni^2+^, and (g) Pb^2+^. For each panel, sample 1: control strain Po1h; sample 2: engineered strain Po1h-3phoX.

Quantitative analysis using a direct reaction method (2 h) showed that the engineered strain achieved significantly higher removal efficiencies than did the control for most metals (Fig. 4a). A time-course study for Co^2+^indicated the reaction reached a plateau after 4 h (Fig. 4b). Kinetic fitting of these Co^2+^ time-course data further showed that the biomineralization process was better described by a pseudo-second-order model (Fig. S5), supporting a progressive precipitation-dominated process rather than instantaneous metal adsorption. Extending the reaction to 4 h improved performance for all metals. The engineered strain achieved removal efficiencies of 79.24% (Cd^2+^), 61.19% (Mn^2+^), 81.24% (Co^2+^), 44.02% (Cr^3+^), 84.57% (Cu^2+^), and 22.84% (Ni^2+^), whereas the control strain showed rates of 29.72%, 26.19%, 25.18%, 14.81%, 70.33%, and 3.87%, respectively (Fig. 4c). The high removal of Pb^2+^(> 98% by both strains) was attributed to the extremely low solubility product (*K*_sp_) of lead phosphate owing to the abiotic precipitation [22,34].

**Fig. 4:**
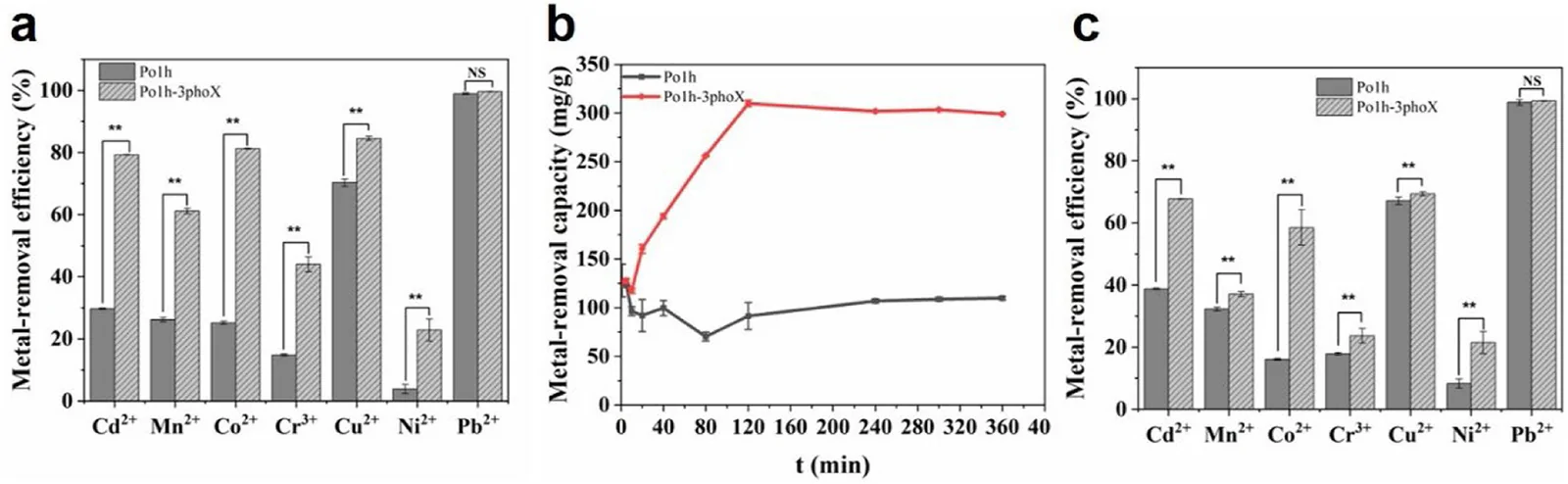
Metal-removal efficiency via the direct reaction method. (a) Removal efficiency for multiple metals after 2 h. (b) Time-course of Co^2+^ removal capacity. (c) Removal efficiency for multiple metals after 4 h. Data are presented as the mean ± SD (*n* = 3). In (a), asterisks indicate statistically significant differences among groups: *P < 0.05, **P < 0.01; NS, not significant (*P* > 0.05) (one-way ANOVA followed by Tukey’s post-hoc test).

To further enhance MIPP efficiency, we designed a stepwise (two-step) reaction method. The rationale was to enable the engineered *Y. lipolytica* to generate sufficient inorganic phosphate from β‑glycerophosphate before exposure to metal ions, thereby avoiding potential metal-induced inhibition of PhoX activity. With this strategy, the cell suspension and the phosphate substrate were pre-incubated for 24 h at 37 °C, followed by addition of metal ions (10 mM final concentration) and an additional 4 h reaction. As shown in Fig. 5, the two-step method dramatically improved metal removal compared with the direct 4 h reaction (Fig. 4c). For instance, Cd^2+^removal increased from 79.24% to 99.23%, and Cr^3+^ removal increased from 44.02% to 90.92% (Fig. 5a). The most pronounced enhancements were observed for metal ions that previously showed moderate removal (e.g., Cr^3+^, Ni^2+^, and Cu^2+^), indicating that the pre‑accumulation of phosphate effectively overcame the kinetic limitations of metal‑phosphate precipitation under direct mixing conditions. Correspondingly, the metal-removal capacities of the engineered strain reached 1251.64 mg Cd^2+^/g biomass, 607.71 mg Mn^2+^/g biomass, 618.38 mg Co^2+^/g biomass, 532.39 mg Cr^3+^/g biomass, 637.17 mg Cu^2+^/g biomass, and 517.87 mg Ni^2+^/g biomass (Fig. 5b). This two-step method presents a highly effective operational strategy for wastewater remediation.

**Fig. 5:**
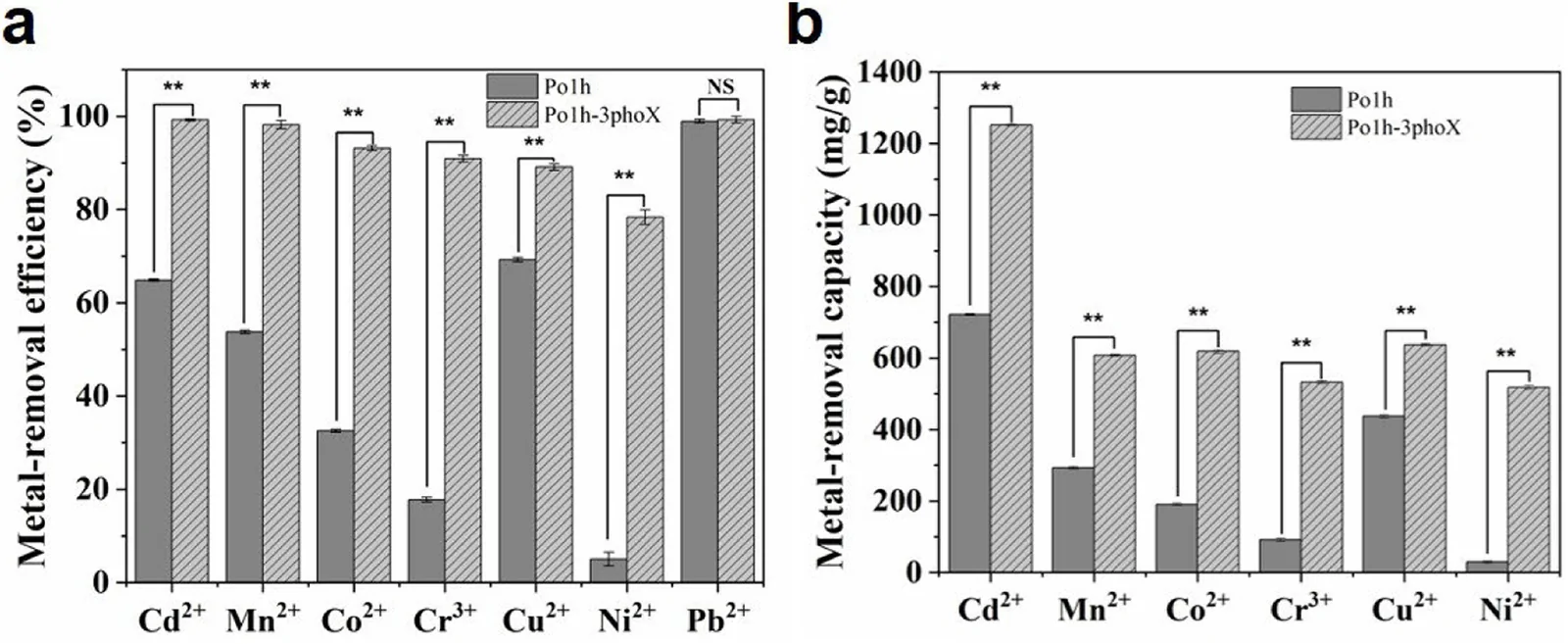
Metal-removal performance via the two-step reaction method (24 h + 4 h). (a) Metal-removal efficiency. (b) Metal-removal capacity. Data are presented as the mean ± SD (*n* = 3). Asterisks indicate statistically significant differences among groups: *P < 0.05, **P < 0.01; NS, not significant (*P* > 0.05) (one-way ANOVA followed by Tukey’s post-hoc test).

The metal-specific removal efficiencies can be rationalized by the solubility product constant (*K_sp_*) values of the corresponding metal phosphates compiled from the literature [[35], [36], [37], [38], [39]]. As shown in Table S2, the *K_sp_* values for Pb_5_(PO_4_)_3_OH, Pb_3_(PO_4_)_2_, Cd_3_(PO_4_)_2_, Co_3_(PO_4_)_2_, Ni_3_(PO_4_)_2_, and Cu_3_(PO_4_)_2_ were 1.70 × 10^−81^, 1.00 × 10^−54^, 2.53 × 10^−33^, 2.05 × 10^−35^, 4.74 × 10^−32^, and 1.40 × 10^−37^, respectively, with the specific references listed in Table S2. Notably, Pb^2+^, showed the lowest *K_sp_*, achieved near-quantitative removal (>99%), whereas Ni^2+^, with a higher *K_sp_* (4.74 × 10^−32^), showed lower removal efficiency (78.35%). For Cr^3+^, the *K_sp_* of CrPO_4_ varied with the hydration state and crystallinity, with literature estimates generally ranging from 10^−17^ to 10^−23^ (see [36], for p*K_sp_* values of different hydrates). This range was consistent with the intermediate removal efficiency of Cr^3+^ (90.92%). Although a reliable *K_sp_* value for Mn_3_ (PO_4_)_2_ was not available in the consulted sources, the high removal efficiency of Mn^2+^ (98.22%) suggests a similarly low solubility. These thermodynamic trends collectively account for the observed metal specific removal hierarchy (Pb^2+^ ≈ Cd^2+^ > Co^2+^ > Ni^2+^ > Cu^2+^ > Cr^3+^).

### 3.4 Morphological and structural analysis of minerals

Advanced microscopy and spectroscopy were employed to characterize the MIPP products. SEM analysis revealed distinct morphologies compared with the control. For Co^2+^, the control strain (Po1h) formed amorphous flakes, whereas the engineered strain (Po1h-3phoX) produced elongated crystals aggregated into large, porous spherical structures (Fig. 6a and 6b). The engineered strain formed metal-specific precipitates. Notably, Cd^2+^, Ni^2+^, and Pb^2+^ generated dense, amorphous coatings that encapsulated cells (Fig. 6c, 6*g*, and 6h). In contrast, Mn^2+^ yielded tightly packed, sheet‑like polymers (Fig. 6d), whereas Cr^3+^ and Cu^2+^produced regular spherical crystalline aggregates (Fig. 6e and 6f). Energy‑dispersive X‑ray spectroscopy (EDS) analysis confirmed the presence of phosphorus, oxygen, and the respective metals in these structures (Fig. S4), verifying their phosphate mineral composition.

**Fig. 6:**
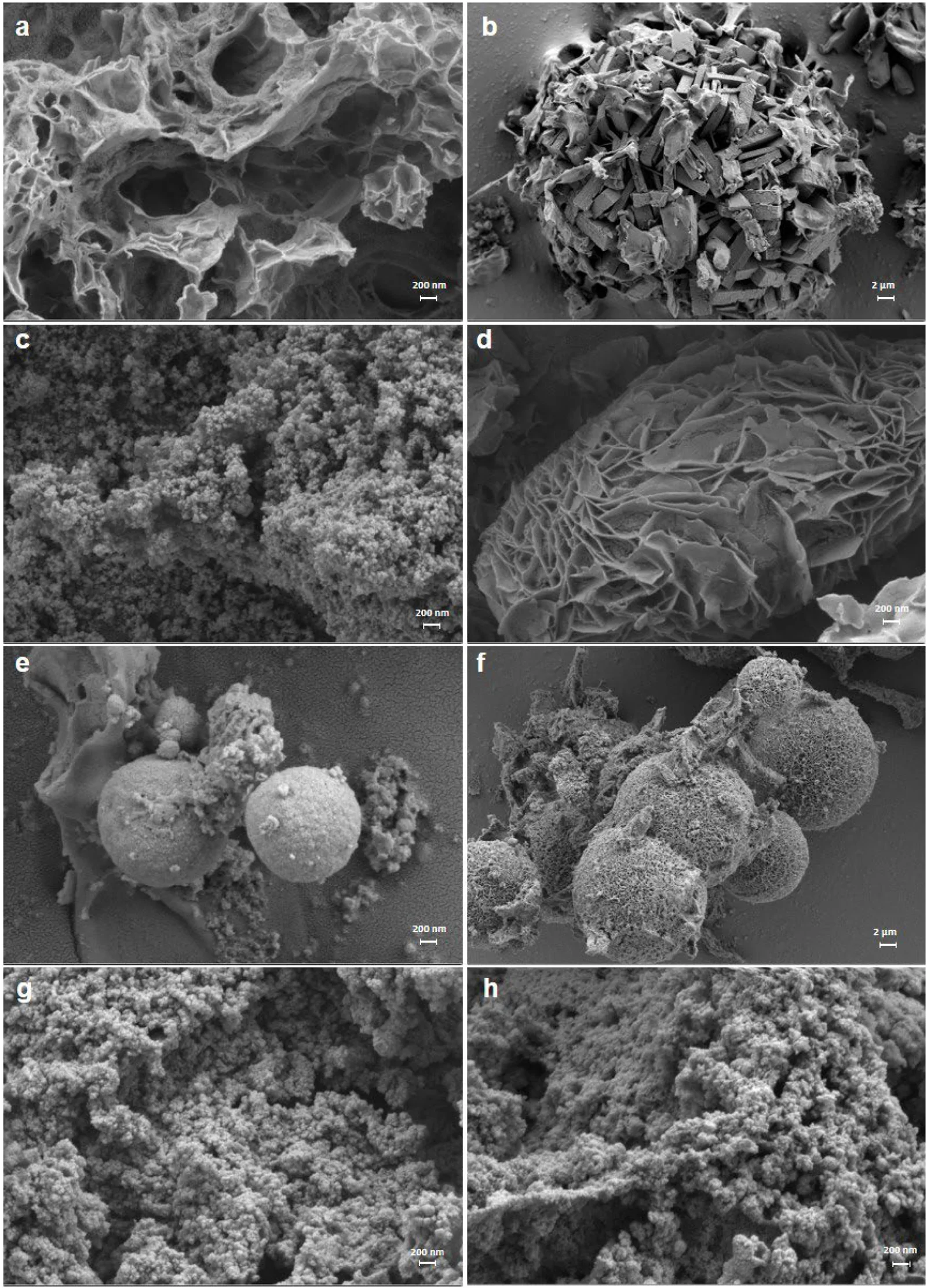
SEM images of MIPP products. (a, b) Co^2+^precipitates. (c) Cd^2+^precipitates. (d) Mn^2+^ precipitates. (e) Cr^3+^precipitates. (f) Cu^2+^precipitates. (g) Ni^2+^precipitates. (h) Pb^2+^ precipitates.

TEM analysis provided critical ultra-structural insights into the cell–mineral interface. For Co^2+^, the engineered strain exhibited a high density of flocculent, surface-associated mineral particles, starkly contrasting with the sparse adsorption observed on control cells (Fig. 7a and 7b). This clear disparity underscored the enzymatic activity’s role beyond native cellular adsorption. Observations of the engineered strain with other metals revealed distinct mineralization patterns consistent with SEM findings. Cd^2+^ biomineralization produced dark, granular aggregates enveloping the cell surface, with substantial intracellular dark aggregates also present, suggesting potential internal mineralization or transport (Fig. 7c). After Mn^2+^exposure, numerous transparent, flake‑like polymers surrounded the cells (Fig. 7d). Cr^3+^ treatment resulted in small, rounded particles adsorbed on the cell surface (Fig. 7e), whereas Cu^2+^ biomineralization generated extensive, outward-growing networks of dark flocculent material (Fig. 7f). Similarly, Ni^2+^ and Pb^2+^ mineralization showed dense adsorption of small particles and the formation of outward-extending flocculent polymers (Fig. 7*g* and 7h).

**Fig. 7:**
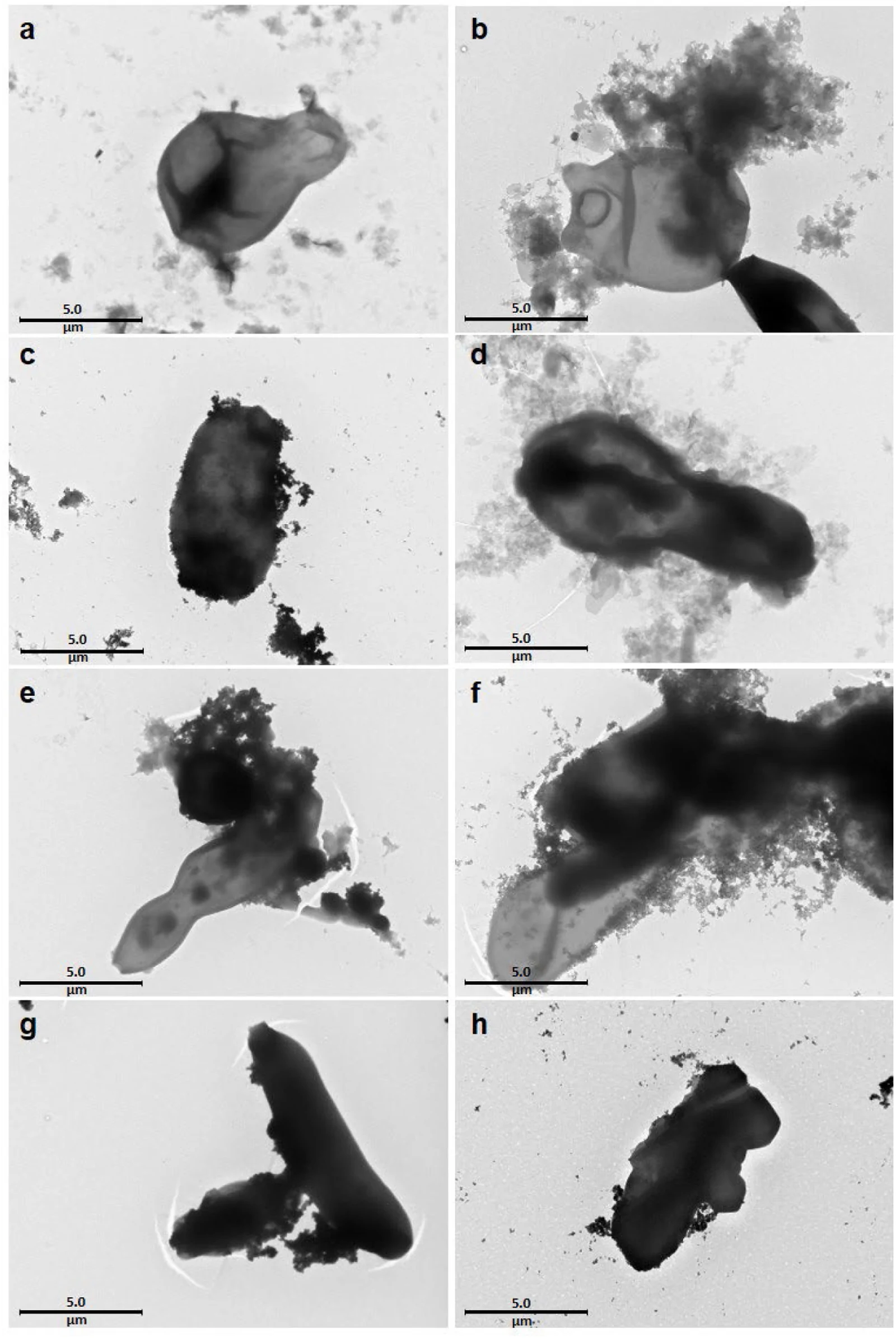
TEM images of MIPP products. (a, b) Co^2+^precipitates. (c) Cd^2+^precipitates. (d) Mn^2+^precipitates. (e) Cr^3+^precipitates. (f) Cu^2+^precipitates. (g) Ni^2+^precipitates. (h) Pb^2+^precipitates.

The strong correlation between the SEM and TEM observations confirmed that the engineered *Y. lipolytica* facilitated the formation of abundant, metal-specific mineral structures—ranging from flakes to spherical particles—that closely associated with the cell surface. Notably, the frequent observation of intracellular dark aggregates across multiple metals, together with EDS data (Fig. S4) confirming the presence of P, O, and the corresponding metals, suggests the possibility of intracellular mineral formation or sequestration. However, we acknowledge that definitive confirmation of intracellular mineralization may require higher-resolution techniques (e.g., cryo-TEM or X-ray absorption spectroscopy) and this represents an interesting direction for future investigation.

#### 3.4.1 Crystallographic and spectroscopic verification of the mineral phase

The mineral phase of the Co^2+^ precipitate was identified as cobalt phosphate octahydrate Co_3_(PO_4_)_2_·8H_2_O by XRD, matching the standard crystalline pattern (PDF #33–0432; Fig. 8a). This finding was confirmed by Fourier-transform infrared spectroscopy, which showed strong peaks for phosphate (PO_4_^3–^) asymmetric stretching (1027–1079 cm^–1^), and bending (571–778 cm^–1^) (Fig. 8b) [40,41], along with bands for crystalline water [42,18]. The intensity of these phosphate peaks was markedly stronger for the engineered strain than for the control, corroborating the enzymatic generation of phosphate leading to stable mineral formation.

**Fig. 8:**
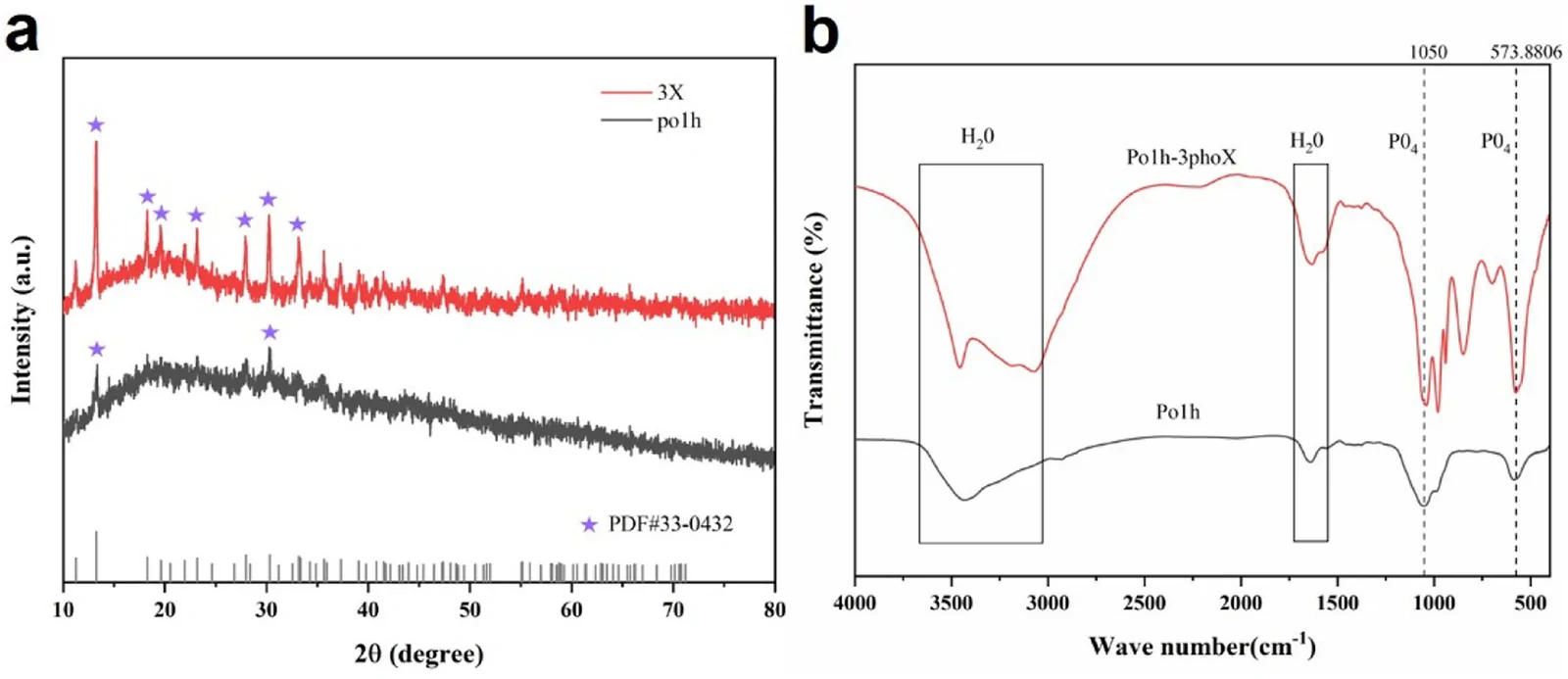
XRD and FT-IR analysis of Co^2+^MIPP products. (a) XRD pattern. (b) FT-IR spectrum.

## 4 Discussion

We demonstrated a microbially mediated pathway for altering the environmental fate of heavy metals in this paper. The engineered *Y. lipolytica* strain served as a biocatalytic platform, secreting PhoX to hydrolyze organophosphates and induce precipitation of soluble metal ions via the formation of stable phosphate minerals. This transformation is environmentally critical, as it shifts metals from a bioavailable, mobile state to a geochemically stable, less toxic mineral phase [[43], [44], [45], [46]]. This process is underscored by high removal capacities and the crystallization of Co_3_ (PO_4_)_2_·8H_2_O, thus showing great potential to significantly reduce metal lability and ecological risk in contaminated systems.

The optimized two-step reaction method developed in this study not only achieved near‑quantitative removal and high capacity for multiple heavy metals but also offers several advantages over conventional remediation technologies. As summarized in Table S3, our PhoX‑based MIPP system exhibited superior performance when compared with conventional MIPP, MICP, chemical precipitation, and biosorption in terms of removal efficiency, capacity, by‑product generation, and long‑term mineral stability [[8], [47], [48], [49]]. Notably, conventional MIPP using *Burkholderia* sp. QY14 required 10 mM Ca^2+^ to achieve 99.97% Cd^2+^ removal, whereas our system achieved 99.23% Cd^2+^ removal without any Ca^2+^ addition [49]. Unlike MICP, which releases ammonia as a toxic by‑product, our MIPP process is completely free of hazardous by‑products. Furthermore, the formation of crystalline metal phosphate minerals ensures superior long‑term environmental stability versus the amorphous sludge generated by chemical precipitation or surface‑limited biosorption. These findings are consistent with earlier reports on biosorption and bio‑precipitation systems [[18], [50]], further underscoring the superior performance of our enzymatic MIPP approach. Kinetic analysis of Co^2+^ removal revealed that the biomineralization process followed a pseudo-second-order model (Fig. S5), indicative of a progressive precipitation driven mechanism rather than instantaneous adsorption. These data support the view that metal removal occurred primarily through enzymatically generated phosphate ions reacting with metal cations to form stable solid phases. Thus, this enzymatic MIPP strategy represents a significant advance in sustainable bioremediation technology. It should be noted that XRD analysis was performed only for the Co^2+^ precipitate (confirming Co_3_ (PO_4_)_2_·8H_2_O); however, EDS analysis confirmed the presence of P, O, and the metals of interest in all other precipitates (Fig. S4), supporting their phosphate mineral nature. Further work will include XRD characterization of Pb^2+^ and Cd^2+^ phosphate minerals.

In this study, co-expression of *vgb, pdi, kar2*, or *hac1* did not improve PhoX production, and co-expression of *pdi, kar2*, or *hac1* even reduced yields. This outcome may be explained by several factors. First, multi-gene overexpression imposes a significant metabolic burden on the host, diverting cellular resources away from recombinant protein synthesis [51,52]. Second, the native secretion machinery of *Y. lipolytica* may already operate near its capacity, so introducing additional chaperones or unfolded protein response (UPR) activators could cause secretion pathway saturation or disturb the endogenous folding equilibrium [53]. Third, co-expression of Hac1, which activates the UPR, may disrupt finely tuned UPR homeostasis and thereby reduce, rather than enhance, PhoX secretion. The lack of enhancement by Vgb suggests that oxygen supply was not limiting under our experimental conditions [[54], [55], [56]]. Collectively, these results indicate that the native secretory pathway of *Y. lipolytica* is sufficient for efficient PhoX production, simplifying strain engineering requirements.

Although the present findings provide proof‑of‑concept at the shake‑flask scale, further scale‑up studies are needed to assess practical applicability. Ongoing work in our laboratory is focused on bioreactor‑scale process development, including the optimization of oxygen transfer, agitation, and continuous operation modes. These efforts, together with techno‑economic analysis, will pave the way for potential industrial implementation of this PhoX-based MIPP system.

## 5 Conclusions

In this study, we engineered a recombinant *Y. lipolytica* strain for high-level secretion of PhoX, establishing an efficient biological pathway for metal immobilization. The strain demonstrated broad-spectrum precipitation of multiple heavy metals via enzymatic phosphate release, with a two-step process enabling near-quantitative removal and high sequestration capacities. Microscopic and crystallographic analyses confirmed the formation of cell-associated, crystalline metal phosphate minerals—exemplified by Co_3_(PO_4_)_2_·8H_2_O (XRD)—with EDS data further supporting the phosphate mineral nature of Cd^2+^, Mn^2+^, Cr^3+^, Cu^2+^, Ni^2+^, and Pb^2+^ precipitates. Kinetic analysis further indicated that a pseudo‑second‑order process occurred (Fig. S5), consistent with a precipitation‑dominated mechanism. By transforming soluble, toxic metal ions into stable mineral phases using a eukaryotic microbial host, this work highlights a novel microbial mechanism for altering metal speciation and reducing environmental bioavailability. The findings contribute to the development of biologically based remediation strategies that target the chemical fate of pollutants, offering a promising approach for mitigating metal mobility and toxicity in contaminated water and soil.

## Data availability statement

The data that support the findings of this study are available from the corresponding author upon request.

## CRediT authorship contribution statement

**Zhen Zheng:** Writing – original draft, Visualization, Validation, Investigation, Data curation. **Jianping Liu:** Writing – review & editing, Visualization, Validation, Formal analysis. **Xiaoman Xie:** Writing – review & editing, Validation, Formal analysis. **Xinyi Hu:** Validation, Formal analysis, Data curation. **Wanlin Zheng:** Writing – review & editing, Formal analysis. **Shuhan Dai:** Validation, Formal analysis. **Qiyang Wang:** Writing – review & editing, Visualization, Formal analysis, Data curation. **Yuan Liao:** Formal analysis, Data curation. **Jinyong Yan:** Writing – review & editing, Visualization, Methodology, Formal analysis. **Min Yang:** Writing – review & editing, Formal analysis. **Yunjun Yan:** Writing – review & editing, Resources, Methodology, Conceptualization. **Li Xu:** Writing – review & editing, Visualization, Supervision, Resources, Methodology, Funding acquisition, Formal analysis, Data curation, Conceptualization.

## Declaration of competing interest

The authors declare that they have no known competing financial interests or personal relationships that could have appeared to influence the work reported in this paper.

## Appendix Supplementary materials

Image, application 1
