# Supplementary material for: Engineered Yarrowia lipolytica for sustainable heavy metal bioremediation via enhanced alkaline phosphatase-driven biomineralization
Source: Eng Microbiol. 2026 Oct 23;6(4):100310. doi: 10.1016/j.engmic.2026.100310 (PMC13770613; doi:10.1016/j.engmic.2026.100310)
Supplement: Supplementary file 1 [file mmc1.docx]

Supplementary Material

**Engineered *Yarrowia lipolytica* for sustainable heavy metal bioremediation via enhanced alkaline phosphatase-driven biomineralization**

Zhen Zheng ^a, #^, Jianping Liu ^a, #^, Xiaoman Xie ^a^, Xinyi Hu ^a^, Wanlin Zheng ^a^, Shuhan Dai ^a^, Qiyang Wang ^a^, Yuan Liao ^b^, Jinyong Yan ^a^, Min Yang ^a^, Yunjun Yan ^a,^ *, Li Xu ^a,^ *

^a^ Key Laboratory of Molecular Biophysics of Ministry of Education, College of Life Science and Technology, Huazhong University of Science and Technology, Wuhan, China;

^b^ Geological Environmental Center of Hubei Province, Hubei Geological Bureau, Wuhan, 430074, China

*Corresponding author:

E-mail addresses: [yanyunjun@hust.edu.cn](mailto:yanyunjun@hust.edu.cn) (Y.J. Yan); [xuli@hust.edu.cn](mailto:xuli@hust.edu.cn) (L. Xu).

^#^Z. Z. and L. J. P. contributed equally to this work.

**Table S1**. Primers used in this study

| **Primers’ name** | **Primers’ sequence (5’-3’)** |
| --- | --- |
| F_pUC57-*phoX*-7166 | CTTTACTATTCTCACGGCCGTTCTGGCCATGTTCAAG |
| R_pUC57-*phoX*-7166 | GTGGGGACAGGCCATGGAATCG ATTTAGTGGTGGTGGTGATGATGCTCA |
| F_*phoX*-2-7166 | ACCGCCTTTACTATTCTCAC |
| R_*phoX*-2-7166 | CAGGCCATGGAATCGATTTA |
| F_*Nsi* I PCR | CAAGTGTAAATCAGTGTTTAATCTCGAA |
| R_*Nsi* I PCR | GACTAGTCTTTAGAATTGGAAGCTGATC |
| F_fuzhu | CACACCACATCACACATACA |
| R_fuzhu | GTAGTAGGAGGCAAGACCGG |

**Table S2**. Solubility product constants (*K_sp_*) of selected metal phosphates at 25°C

| Metal ion | Phosphate mineral | *K_sp_* | | References |
| --- | --- | --- | --- | --- |
| Pb^2+^  Pb^2+^ | Pb_5_(PO_4_)_3_OH  Pb_3_(PO_4_)_2_ | 1.70×10^-81^  1.00 ×10^-54^ | | [66]  [30,46] |
| Cd^2+^ | Cd_3_(PO_4_)_2_ | 2.53×10^-33^ | | [4] |
| Co^2+^ | Co_3_(PO_4_)_2_ | 2.05×10^-35^ | | [4] |
| Ni^2+^ | Ni_3_(PO_4_)_2_ | 4.74×10^-32^ | | [4] |
| Cu^2+^ | Cu_3_(PO_4_)_2_ | 1.4×10^-37^ | | [4] |
| Cr^3+^ | CrPO_4_ (various hydrates) | 10^-17^–10^-23^ (depending on hydration state and crystallinity) | | [17] |
| Mn^2+^ | Mn_3_(PO_4_)_2_ | | Not available |  |

^*^ Note: For Pb_3_(PO_4_)_2_, the *K_sp_* value is widely cited in standard reference handbooks [30,46]. According to ChemicalBook (2026), the *K_sp_* of anhydrous green CrPO_4_ is 2.40 × 10^-23^, while that of violet hexahydrate CrPO_4_·6H_2_O is 1.00 × 10^-17^. The range of 10^-17^ to 10^-23^ reflects this variation within hydration state and crystallinity.

**Table S3.** Comparison of heavy metal-removal performance among different remediation methods used

| Method | Target metal | Removal efficiency (%) | Removal capacity (mg/g) | Key advantage/disadvantage | References |
| --- | --- | --- | --- | --- | --- |
| This work (PhoX‑MIPP, stepwise) | Cd^2+^/ Pb^2+^ | 99.23/ 99.28 | 1251.64/- | No Ca^2+^ needed; crystalline product | - |
| Conventional MIPP | Cd^2+^ | 76.14 (99.97 with Ca^2+^) | – | Requires Ca^2+^ addition | [61] |
| MICP | Pb^2+^/Cd^2+^ | 90–98 | – | Produces NH_3_ by-product | [41] |
| Chemical precipitation | Cd^2+^/Zn^2+^ | 95–99.9 | – | Generates hazardous sludge | [43] |
| Biosorption | Cd^2+^ | – | 22.4–56.5 | Capacity limited by surface sites | [22] |

Table footnote: “–” indicates not reported or not applicable. For conventional MIPP, the value in parentheses (99.97%) was achieved with the addition of 10 mM Ca^2+^, our system requires no Ca^2+^ addition. For biosorption, removal efficiency is highly dependent on experimental conditions; the reported capacity range is derived from the maximum biosorption capacities of three different microbial strains [22].

**Table S4.** Extracellular protein- and biomass-normalized PhoX activity of optimized multi-copy strains

| Strain | OD_600_ | ECP (mg/mL) | PhoX activity (U/mL) | U/mg ECP | U/OD_600_ |
| --- | --- | --- | --- | --- | --- |
| Po1h-1phoX | 38.50 ± 2.29 | 0.28 ± 0.09 | 8.12 ± 0.32 | 31.25 ± 8.46 | 0.21 ± 0.02 |
| Po1h-2phoX | 33.33 ± 2.25 | 1.29 ± 0.38 | 53.57 ± 3.22 | 43.85 ± 12.57 | 1.61 ± 0.14 |
| Po1h-3phoX | 33.00 ± 3.77 | 2.34 ± 0.20 | 124.12 ± 5.14 | 53.30 ± 6.03 | 3.80 ± 0.53 |
| Po1h-4phoX | 34.17 ± 3.88 | 1.14 ± 0.10 | 64.34 ± 2.85 | 56.72 ± 7.37 | 1.89 ± 0.14 |

Note: Values are presented as mean ± SD from three independent biological replicates. ECP, extracellular protein concentration. The medium blank represents the protein concentration of the undiluted uninoculated medium. ECP was calculated as protein assay value × protein dilution factor − medium blank. U/mg ECP represents protein-normalized activity of the crude extracellular preparation rather than the specific activity of purified PhoX.

**Sequence S1.** *phoX* gene sequence

GGCCGTTCTGGCCATGTTCAAGCGAGTCGCCGCCGCTCTGCTGCTTGCTTCTGCTATGCCCGTCATGGCCCAGACCCCTGCTCCTGCTCCTGTTGCTGCTACCGTGGCTCCTGTCGCTGCTCCTAAGCTGATCGTCGCCATCTCCATCGACCAGTTCTCCGCCGATCTGTTCTCCGAGTACCGACAGTACTACTCCGCCGGCCTGAAGCGACTGACCCAGGGTGCTGTTTTTCCCCGAGGCTACCAGTCCCACGCCGCTACTGAGACTTGCCCTGGTCATTCCACCATCCTGACCGGCTCCCGACCTGCTCGAACTGGTATCATCGCCAACTCCTGGTTTGATCTGGGCACCGGCCGAGAGGACAAGGCTATTTACTGCGCCGAGGACGAGTCCGCCCCTGGTTCTAACTCCGACGCTTACGTCGCCTCCCCCGTTCATCTGAAGGTCCCTACTCTGGGCGGCCGAATGAAGGCTGCTAACCCTGCTACCCGAGTCGTTTCCGTGGCCGGTAAGGACCGAGCTGCTATCATGATGGGCGGTCCCACCGCTGATCAGGTCTGGTGGCTTGGAGGCGCTCAGGGTTACGTCTCCTACAAGGGCGTTCCTACCCCTCCCCTGGTGGCTAAGGTGAACCAGGTGTTTGCCCAGCGACTTGCCCAGCCTAACCCCGGTTTTGAGCTGCCTGCTCAGTGTGCCGCTAAGGATTTCCCCGTCCAGGCCGGTAACCGAACCGTTGGTACCGGACGATTCGCCCGAGACGCTGGTGATTACAACGGCTTCCGAATCTCCCCCGAGCAGGACGCTATGACCCTGGCTTTCGCTGCCGCTGCTATCGAGACTATGGGCCTCGGTAAGCAGACTCAGACCGACATTATTTCCATCGGCCTCTCCGCTACCGACTACATTGGCCACACCTACGGTACCGAGGGCACTGAGTCCTGTATTCAGGTCGATCGACTGGATCAGGAGCTGGGCGCTTTCTTCGACCGACTGGACAAGGATGGCATCGACTACGTGGTCGTCCTGACCGCTGACCACGGAGGTCATGACCTGCCTGAGCGACACCGACTGAACGCTATGCCCATGGAGTCTCGAGTCGATATGGCCCTTACCCCCAAGGCCCTGTCCGCTGCTGTTGCTGAGAAGGCTGGACTGCCCGGTCGAAAGGTTATCTGGGGCGACGGTCCTGCCGGTGATCTTTACTTTGACAAGGGTCTGACCGCCGCCCAGCGAGCTCGAGTTGAGGCTGAGGCTCTGAAGCTGCTGCGAGCTCACCCTCAGGTCGAGACTGTGTTCACCAAGGCCGAGATTGCCGCCACCCCTTCTCCTTCTGGCCCTCCTGAGTCCTGGTCCCTTATTCAGGAGGCCCGAGCCTCCTTTGACTCCGAGCGATCTGGTGACCTGCTGCTGCTCCTGAAGCCCCGAGTTATGTCCATCCCCGAGCGAGCTGTCATGGGCGCTGTTGCTACCCACGGTTCCCCTTGGGACACCGATCGACGAGTCCCTATCCTTTTCTGGCGAAAGGACATGCGACACTTTGAGCAGCCCCTTGGCGTCGAGACCGTCGATATCATGCCCTCCCTGGCCGCTCTGATCGGTCTTCCTGTCGCTAAGGGCGAGATCGACGGCCGATGTCTGGACCTTATCGCCGGTGCTGGAGACTCCTGCGCTCCTGAGCATCATCACCACCACCACATCGAT

**Fig. S1.**

**Fig. S1.** Mass spectrometric identification of the recombinant Po1h-3phoX protein. Peptide sequences identified by mass spectrometry are highlighted in red on the protein sequence.

**Fig. S2.**

**Fig. S2.** Relative enzyme activity of the recombinant strain Po1h-3phoX co-expressing different auxiliary proteins

**Fig. S3.**

**Fig. S3.** Co^2+^-removal efficiency by the engineered Po1h-3phoX strain in media with and without phosphate buffer

**Fig. S4.**

**Fig. S4.** EDS analysis of metal-containing precipitates produced by MIPP. The products are (a) Co^2+^, (b) Cd^2+^, (c) Mn^2+^, (d) Cr^3+^, (e) Cu^2+^, (f) Ni^2+^, and (g) Pb^2+^ precipitates.

**Fig. S5.**

**Fig. S5.** Linearized pseudo-second-order kinetic model for Co^2+^ biomineralization by the recombinant strain Po1h-3phoX
